# Impact of income on the profile of cardiovascular risk factors among hypertensives in a Nigerian tertiary health centre: a cross-sectional study

**Published:** 2009-08

**Authors:** KM Karaye, BN Okeahialam, SS Wali

**Affiliations:** Department of Medicine, Bayero University and Aminu Kano Teaching Hospital, Kano, Nigeria; Department of Medicine, Jos University Teaching Hospital, Jos, Nigeria; Department of Medicine, Murtala Muhammad Specialists’ Hospital, Kano, Nigeria

## Abstract

**Background:**

In most developed countries, risk factors for cardiovascular diseases (CVD) are more prevalent in low socio-economic classes. However, the pattern in developing countries appears to be different. This study sought to evaluate and compare risk factors for CVD as well as absolute CVD risk in hypertensive subjects grouped by income in Kano, Nigeria.

**Methods:**

The study was cross-sectional in design and carried out in Aminu Kano Teaching Hospital, Kano, Nigeria. Seventy treatment-naïve hypertensives and an equal number of hypertensives on treatment were recruited by balloting from the outpatient clinics, and then regrouped into low- and high-income earners. These two groups were then compared in terms of their profile of CVD risk factors and absolute CVD risk. All the assessed CVD risk factors are recognised in standard guidelines for the management of persons with systemic hypertension.

**Results:**

The low-income group comprised 45 patients (32.1%) while the remaining 95 (67.9%) had a high income. The most prevalent CVD risk factor was dyslipidaemia, found in 77.8 and 71.6% of low- and high-income earners, respectively (*p* = 0.437). The prevalence of proteinuria was significantly higher among low-income earners (42.2%) compared with highincome earners (15.8%) (*p* = 0.001). Mean serum creatinine was also higher among low-income earners but the difference did not reach statistical significance (*p* = 0.154). Very high CVD risk was found in 75.6 and 70.5% of low- and high-income earners, respectively (*p* = 0.535).

**Conclusion:**

Dyslipidaemia and very high CVD risk were found in over 71% of the patients regardless of their level of income. Low-income earners had a higher prevalence of indices of renal damage. These findings pose a great challenge to the present and future management of all subjects, particularly those in the low-income group, given that in Nigeria, healthcare is largely paid for directly out of their pockets.

## Summary

In most developed countries, risk factors for coronary artery disease and other cardiovascular diseases (CVD) are more prevalent in groups of lower socioeconomic status.^1^ The pattern in developing countries appears to be different. In the INTERHEART African study, for example, risk factors for acute myocardial infarction were found to be more prevalent in black Africans with high income and education levels than among those with a lower income.^2^ This pattern is typical for a population in epidemiological transition, and the findings parallel those in European countries in the early 20th century, at the beginning of the CVD epidemic.^3^ However, as healthcare improves for the wealthy, there will be a reversal in this trend, and the poorer and more disadvantaged people will suffer the larger burden of CVD. This is especially true for risk factors of CVD, which are predictors for future events.^4^ The INTERHEART study also showed that systemic hypertension had a greater impact on the risk of acute myocardial infarction among black Africans than among other racial groups.^2^ In the INTERHEART study, however, more than 80% of the subjects were South Africans, and only 13 were recruited from Nigeria.^2^

We therefore sought to evaluate and compare risk factors for CVD as well as absolute CVD risk in hypertensive subjects grouped by income in Kano, Nigeria.

## Methods

The study was carried out in Aminu Kano Teaching Hospital, Kano, Nigeria. The hospital is the only tertiary health centre in the most populous Nigerian State of Kano, north-western Nigeria. It receives referrals from hospitals in Kano and Jigawa states, as well as from the neighbouring states of Katsina, Yobe and Bauchi. In the hospital, patients pay for services directly out of their pockets, though a minority enjoy the National Health Insurance Scheme where the cost of services is subsidised.

The Research Ethics Committee of Aminu Kano Teaching Hospital reviewed and approved the study protocol. All recruited patients gave written informed consent to participate in the study, which conformed to the principles outlined in the Declaration of Helsinki on the ethical principles for medical research involving human subjects.^5^

The study was cross-sectional in design. A minimum sample size of 63 patients was calculated using a validated formula,^6^ applying a precision of 10% and prevalence of hypertension of 20%. This prevalence of 20% was extrapolated from the 1997 report of the Committee on Non-Communicable Diseases in Nigeria.^7^ One hundred and forty patients were recruited. In the initial design, 70 of these patients were on drug treatment for hypertension while the other 70 were treatment-naïve. The two groups were matched for age and gender. Ten patients in each group were selected from their cohort on a weekly clinic day, by balloting, for seven weeks. The profile of CVD risk factors, absolute CVD risk and serum cholesterol for the two hypertensive groups have been previously assessed and compared with a non-hypertensive control group, and the findings published.^8^, ^9^

In the present study, the 140 recruited hypertensive subjects were regrouped into two, based on their level of monthly income; those with a low monthly income (low-income group) and those with a higher monthly income (high-income group). These two groups were then compared in terms of their profile of CVD risk factors and absolute CVD risk.

Low income was defined as having a monthly income of less than 10 000 Naira ($77) for a single person, or less than 15 000 Naira ($115) for a couple, and less than an additional 5 000 Naira ($38.50) for each child. This was based on the local purchasing power of the Naira and the salary scale of the Kano state Civil Service in 2004. All the CVD risk factors assessed were recognised in the 2003 World Health Organisation/International Society of Hypertension (WHO/ISH) guidelines for the management of hypertension.^10^ The definitions of the following risk factors were adopted from the above-mentioned guidelines: increased age, family history of premature CVD, sedentary lifestyle, and grading of hypertension.^10^ Hypertension was therefore graded as follows: grade I: systolic blood pressure (SBP) of 140–159 mmHg and/or diastolic blood pressure (DBP) of 90–99 mmHg; grade II: SBP of 160–179 mmHg and/or DBP of 100–109 mmHg; and grade III: SBP ≥ 180 mmHg and/or DBP ≥ 110 mmHg. History of duration of hypertension was obtained from the patient, estimated from the first time he/she became aware of the hypertension. A history of tobacco smoking was considered a risk factor if smoking was daily, regardless of the number of cigarettes. The diagnosis of diabetes mellitus (DM) was based on WHO criteria.^11^

Left ventricular hypertrophy (LVH) was determined using Araoye’s code system for LVH on a 12-lead electrocardiogram (ECG) in the hypertensive patients.^12^ This has been shown to have a higher sensitivity and specificity than other methods for the ECG determination of LVH in black Africans with systemic hypertension.^13^ Urine analysis for protein was done using commercial reagent strips (Combi 2 Medi-Test, by Macherey-Nagel, Germany). CVD risk (typical 10-year risk of stroke or myocardial infarction) was stratified as low (< 15% risk), medium (15–20% risk), high (20–30% risk) and very high (> 30% risk), based on the recommendations of the WHO/ISH.^14^ Dyslipidaemia was defined by the presence of any of high total cholesterol (TC) (> 5.2 mmol/l), high low-density lipoprotein cholesterol (LDL-C) (> 3.38 mmol/l) or low high-density lipoprotein cholesterol (HDL-C) (< 1.0 mmol/l). These cut-off values are based on the Adult Treatment Panel III (ATP III) recommendations for borderline-high total cholesterol (TC) and LDL-C, and low HDL-C, respectively.^15^ Renal failure was simply defined as the presence of serum creatinine concentrations of ≥ 176 μmol/l (≥ 2 mg/dl).^10^

Data were analysed with SPSS version 11.5. Means and standard deviations were computed for quantitative variables and the Student’s *t*-test was used to compare means. The Chi-squared or Fisher’s exact tests were used to test for significance among categorical variables. The binary logistic regression model and correlation coefficient were used to analyse the association between any outcome of interest and a number of variables. A *p*-value of < 0.05 was considered significant.

## Results

Data were collated over seven weeks, between April and May 2004. The low-income group comprised 45 patients (32.1%) while the remaining 95 (67.9%) had a high income. Table 1 compares the CVD risk factors and CVD risk of the two groups. It shows that the prevalence of dyslipidaemia was above 71% in both groups, without significant statistical difference between them (*p* = 0.437). The prevalence of a sedentary lifestyle was also high and above 57%, while that of increased BMI was above 53% in both groups, but the differences between the groups did not reach statistical significance (*p* > 0.05). As the groups were originally matched for age and gender, differences in their mean age, and prevalence rates of increased age and male gender were not statistically significant.

Table 1 also shows that the prevalence of proteinuria was significantly higher among low-income earners compared with high-income earners (*p* = 0.001). Mean serum creatinine, and mean systolic and diastolic blood pressures (SBP and DBP, respectively) were also higher among low-income earners but the differences did not reach statistical significance (*p* = 0.154, 0.414 and 0.122, respectively). Table 1 also shows higher levels of mean body mass index (BMI), low HDL-C and triglycerides, and prevalence rates of increased BMI, sedentary lifestyle, DM and smoking among high-income earners compared with low-income earners, but the differences were not statistically significant (*p* ≥ 0.05).

**Table 1:** Baseline Characteristics, CVD Risk Factors And Absolute Risk

	*Low-income earners n = 45 (32.1%)*	*High-income earners n = 95 (67.9%)*	p*-value*
Mean age (SD) (years)	49.93 (12.07)	50.83 (11.92)	0.679
Increased age (%)	9 (20)	23 (24.2)	0.580
Male gender (%)	22 (48.9)	42 (44.2)	0.604
Mean BMI (kg/m^2^)	25.78 (4.56)	27.13 (5.01)	0.128
Increased BMI (%)	24 (53.3)	57 (60.0)	0.456
Mean SBP (mmHg)	164.23 (32.05)	159.85 (28.66)	0.414
Mean DBP (mmHg)	104.62 (20.99)	99.20 (18.36)	0.122
Duration of hypertension (SD) (years)	2.8 (5.70)	4.0 (6.54)	0.294
Dyslipidaemia (%)	35 (77.8)	68 (71.6)	0.437
Mean TC (SD) (mmol/l)	5.45 (1.16)	5.45 (0.96)	0.973
Mean HDL (SD) (mmol/l)	1.34 (0.51)	1.30 (0.47)	0.657
Mean LDL (SD) (mmol/l)	3.55 (1.03)	3.51 (1.04)	0.829
Mean TG (SD) (mmol/l)	1.26 (0.49)	1.32 (0.51)	0.517
Mean serum creatinine (SD) (μmol/l)	126.64 (175.33)	95.99 (78.44)	0.154
Mean FPG (mmol/l)	4.95 (0.64)	4.86 (0.78)	0.525
Smoking (%)	2 (4.4)	7 (7.4)	0.225
Sedentary lifestyle (%)	26 (57.8)	58 (61.2)	0.712
Family history of CVD (%)	7 (15.6)	10 (10.5)	0.395
DM (%)	1 (2.2)	6 (6.3)	0.229
LVH (%)	22 (48.9)	46 (48.4)	0.959
Proteinuria (%)	19 (42.2)	15 (15.8)	0.001*
CVD risk			
Low (%)	0	2 (2.1)	–
Moderate (%)	2 (4.4)	6 (6.3)	0.656
High (%)	9 (20.0)	20 (21.1)	0.886
Very high (%)	34 (75.6)	67 (70.5)	0.535
Hypertension: grades			
I (%)	16 (35.6)	22 (23.2)	0.123
II (%)	6 (13.3)	30 (31.6)	0.021*
III (%)	20 (44.4)	36 (37.9)	0.460

*p-value statistically significant; SD, standard deviation; FPG, fasting plasma glucose.

Mean total cholesterol and the prevalence of left ventricular hypertrophy were almost similar in the two groups. More than 71% of patients in both groups had very high CVD risk, slightly higher among low-income earners but the difference was not statistically significant (*p* = 0.535). Controlled hypertension (SBP/DBP < 140/< 90 mmHg respectively) was found among seven high-income earners (7.4%) and three low-income earners (6.7%) but the difference was not statistically significant (*p* = 0.880). The majority of patients in both groups had grade III hypertension, with a higher prevalence among low-income earners compared with high-income earners, but the difference was not statistically significant (*p* = 0.460). The mean duration of hypertension for all patients was 3.61 ± 6.29 years, and the difference between the low- and high-income earners did not reach statistical significance (*p* = 0.294).

In a logistic regression model controlling for other confounding factors, independent predictors of proteinuria were low income [odds ratio (OR) = 7.52, *p* = 0.001], grades of hypertension (OR = 1.96, *p* = 0.04) and DM (OR = 18.31, *p* = 0.013), while unit rise of serum triglycerides (OR = 0.13, *p* = 0.001) and serum creatinine concentrations (OR = 0.99, *p* = 0.033) were negatively associated with proteinuria (Table 2).

**Table 2:** Independent Predictors Of Proteinuria (In A Logistic Regression Controlling For Potential Confounders)

*Variable*	*Odds ratio*	*95% CI*	p*-value*
Low income	7.52	2.27–24.92	0.001*
Grade of hypertension	1.96	1.03–3.72	0.040*
BMI (kg/m^2^)	1.03	0.89–1.18	0.710
Systolic BP (mmHg)	1.01	0.98–1.04	0.613
Diastolic BP (mmHg)	1.00	0.95–1.04	0.866
Duration of hypertension	1.01	0.92–1.11	0.797
Total cholesterol (mmol/l)	0.51	0.12–2.19	0.365
HDL cholesterol (mmol/l)	0.69	0.16–3.02	0.618
LDL cholesterol (mmol/l)	3.55	0.86–14.62	0.080
Triglycerides (mmol/l)	0.13	0.04–0.43	0.001*
Serum creatinine (μmol/l)	0.99	0.97–1.00	0.033*
CVD risk	0.47	0.16–1.40	0.174
Diabetes mellitus	18.31	1.84–182.42	0.013*

*p-value statistically significant.

Table 3 describes the correlations between proteinuria and several assessed variables. It shows that there was a significant positive correlation between proteinuria and income, grades of hypertension, LVH, and serum levels of LDL cholesterol and triglycerides. It also shows that there was a significant negative correlation between proteinuria and serum creatinine concentrations. However, there was no correlation between proteinuria and duration of hypertension, total and HDL cholesterol, systolic and diastolic blood pressures, and diabetes mellitus. Further analysis showed that serum creatinine correlated negatively with duration of hypertension (*r* = –0.232, *p* = 0.006) and LVH (*r* = –0.274, *p* = 0.001).

**Table 3:** Correlates Of Proteinuria

*Variables*	*Correlation coefficient*	p*-value*
Income	0.288	0.001*
Grades of hypertension	0.178	0.036*
Duration of hypertension	0.062	0.467
Serum creatinine	–0.174	0.04*
LVH	0.249	0.003*
Systolic BP	0.043	0.614
Diastolic BP	0.111	0.194
Diabetes mellitus	0.023	0.788
Total cholesterol	0.008	0.925
LDL cholesterol	0.190	0.025*
HDL cholesterol	–0.086	0.312
Triglycerides	–0.248	0.003*

*p-value statistically significant.

## Discussion

In this study, we assessed the effects of income on the profile of CVD risk factors and on absolute CVD risk among hypertensives in the only tertiary health centre in Kano. In the study centre, there were fewer hypertensives with a low income (32.1 vs 67.9%), perhaps because the costs of services are relatively higher than in other government-owned health centres in the state. It is therefore possible that the majority of low-income earners preferred to go to health centres with cheaper services, and only presented to the more expensive teaching hospital for special reasons such as proximity to their homes or if they were sponsored. Findings in the present study might not be influenced by referral bias, as suggested in our recent study carried out in Murtala Muhammad Specialists’ Hospital, which is a government-owned hospital where costs of services are almost free, and which is patronised by low-income earners in Kano.^16^

The most prevalent CVD risk factor among the studied subjects was dyslipidaemia, found to be higher among low-income earners (77.8%) compared with high-income earners (71.6%), although the difference was not statistically significant (*p* = 0.437). The second and third most common risk factors, sedentary lifestyle and increased BMI, respectively, however tended to be more common among high- than low-income earners (*p* > 0.05). These three common risk factors are known to be related to each other in a vicious circle; one could lead to or potentiate the other. Although high-income earners were heavier than low-income earners (mean BMI 27.13 ± 5.01 vs 25.78 ± 4.56 kg/m^2^) and lived a more sedentary lifestyle, we deduced that their lower prevalence of dyslipidaemia was probably because they were able to eat a relatively healthier diet containing less cholesterol. Unfortunately, we did not assess the dietary habits of the subjects. However, our hypothesis is plausible, given the easier access to dietary information on the Internet and other sources of information accessible to the rich in a developing society like Kano in Nigeria. The financial strength of the high-income earners would also afford them healthier food choices rich in fresh fruits and vegetables, thereby empowering them to put their nutritional knowledge into practice.

The types of foods consumed by individuals have a direct relationship with their pattern of serum lipids. This was demonstrated in a previous study in Benin city, Nigeria, in which senior civil servants with a high income were compared with their junior colleagues with low incomes for the relationship between pattern of food intake and CVD risk factors.^17^ Subjects with high incomes were found to consume more meat and other animal fats, and to have higher BMI, blood pressures, insulin and prevalence of dyslipidaemia, compared with the junior workers. They found that the dietary differences in the consumption of meat, milk and eggs between the junior and senior workers were reflected in increased total fatty acids and arachidonic acid in particular.17 In the Benin study therefore, the disparities between the groups were caused by the different dietary patterns and not by income *per se*, which is in agreement with our hypothesis.

Overall, the subjects were a hypertensive population with poor blood pressure control (in only 14.1%), and the majority had grade III hypertension at the time of evaluation. Given this finding and the high prevalence of other important CVD risk factors among the subjects, it did not come as a surprise that 75.6 and 70.5% of low- and high-income earners had very high CVD risk, with high prevalence rates of end-organ damage, including LVH (> 48% in both groups) and proteinuria. Other workers in Nigeria have recently reported a similar trend among hypertensives.^18^,^19^

It is known that at any given time, different countries or even regions within a country are at different stages of epidemiological transition. This transition can occur not only between different disease categories, but also within a specific disease entity.^20^ This fact was spelled out in the last nationwide non-communicable diseases survey in Nigeria, carried out in 1996, where the people of Kano were found to have the highest prevalence of hypertension and the highest serum concentrations of cholesterol.^7^ More recently, our group reported a high prevalence of dyslipidaemia (70.4%) among patients with stroke in Kano, and 79.0% of the subjects had hypertension.^21^ Therefore, the theory of epidemiological transition is quite real and advancing in Kano in particular, and Nigeria generally.

Both micro- and macroproteinuria are well-recognised risk factors for cardiovascular morbidity and mortality worldwide.^22^ The relationship between income and proteinuria among hypertensive subjects has not been described in our locality. In the present study on hypertensives, prevalence of proteinuria (on dipstick) was found to be significantly higher among lowincome earners (42.2%), compared with patients of high income (15.8%) (*p* = 0.001). In addition, low-income earners tended to have higher mean serum concentrations of creatinine (*p* = 0.154, not significant).

In the logistic regression model controlling for other confounders, independent predictors of proteinuria were low income (OR = 7.52, *p* = 0.001), grades of hypertension (OR = 1.96, *p* = 0.04) and DM (OR = 18.31, *p* = 0.013), while unit rise of serum triglycerides (OR = 0.13, *p* = 0.001) and serum creatinine concentrations (OR = 0.99, *p* = 0.033) were negatively associated with it. Correlates of proteinuria were income, grades of hypertension (and not SBP or DBP), LVH, and serum concentrations of creatinine, LDL cholesterol and triglycerides. Although duration of hypertension did not correlate with proteinuria, it negatively correlated with serum creatinine (correlation coefficient: 0.0232; *p* = 0.006). We have previously reported that in this cohort of patients, proteinuria and increased serum creatinine tended to be higher among treatment-naïve hypertensives with higher BPs compared with those on treatment (and lower BPs), but the differences were not statistically significant (*p* > 0.05).^8^

Overall, the results suggest that the higher the BPs the worse the renal function, and secondly that hypertensive patients generally presented to the hospital after renal damage had already occurred. Thirdly, the duration of hypertension as estimated by the patients was therefore likely to be inaccurate.

From the foregoing, we have shown that indices of early renal disease were more common among hypertensives with a low income. These low-income earners also tended to have higher mean blood pressures and prevalences of dyslipidaemia, which are among the predictors of the proteinuria. These findings are relevant in several ways. Firstly, it is known that in Nigeria, hypertension is more common in both extremes of socio-economic classes – the very rich and the very poor.^7^ It has also been reported that over 70% of Nigerians are poor, living below the poverty line.^23^ In addition, it is common knowledge that the majority of Nigerians pay for healthcare directly out of their pockets. Even the few Nigerians who enjoy the recently introduced National Health Insurance Scheme would have to pay directly for certain medical services including renal replacement therapy (such as dialysis).^24^

It is therefore clear that strategies to reverse the indices of renal dysfunction must be implemented to prevent their progression to end-stage renal disease (ESRD). This is because the management of ESRD is expensive, and the costs of management cannot be met by most individuals and states in the developing world, including Nigeria. In the study centre at the time of writing this article, three sessions of haemodialysis cost between 31 000 and 38 000 Naira ($269.50–319.33). Clearly, only a few patients can afford to initiate or sustain the dialysis. Unfortunately, it is estimated that by the year 2010, the global cost of maintenance haemodialysis will rise to the enormous sum of $1.1 trillion.^25^ Hence prevention of chronic kidney disease is virtually our only affordable option.

Given the cross-sectional design of this study, the findings need to be further confirmed in a large prospective study. The strength of this study is the fact that it is the first in our locality to show significant positive correlations between proteinuria and low income in hypertensives, among other findings. The weaknesses include the small sample size, particularly in the low-income group, and lack of data on the dietary habits of the subjects.

## Conclusion

Dyslipidaemia and very high CVD risk were found in over 71% of the patients regardless of their level of income. Low-income earners had a higher prevalence of indices of renal damage. These findings pose a great challenge to the present and future management of all the subjects, particularly those in the low-income group, given the fact that in Nigeria healthcare is largely paid for directly out of the patient’s pocket.
